# Microsampling in toxicology studies – maximising the scientific, business and 3Rs advantages

**DOI:** 10.1093/toxres/tfaf045

**Published:** 2025-03-31

**Authors:** Helen Prior, Adeyemi O Adedeji, Romalie Allen, Derek Angus, Daniel Baker, Hollie Blunt, David Coleman, Helen-Marie Dunmore, Elisa Passini, Tara Putnam, Marie-Luce Rosseels, Neil Spooner, Jane Stewart, Carol Strepka, Alan Stokes, Tom Verhaeghe, Amanda Wilson, Fiona Sewell

**Affiliations:** National Centre for the Replacement, Refinement and Reduction of Animals in Research (NC3Rs), 215 Euston Road, London, NW1 2BE, United Kingdom; Genentech Inc, 1 DNA Way, South San Francisco, CA, 94080, United States; Labcorp, Otley Rd, Harrogate, HG3 1PY, United Kingdom; Syngenta, Jealotts Hill Research Station, Bracknell, RG42 6EY, United Kingdom; AstraZeneca,1 Francis Crick Avenue, Cambridge, CB2 0AA, United Kingdom; Sequani Ltd, Bromyard Road, Ledbury, HR8 1LH, United Kingdom; Labcorp, Woolley Road, Alconbury, Huntingdon, PE28 4HS, United Kingdom; Charles River Laboratories, Elphinstone, Tranent, EH33 2NE, United Kingdom; National Centre for the Replacement, Refinement and Reduction of Animals in Research (NC3Rs), 215 Euston Road, London, NW1 2BE, United Kingdom; Boehringer Ingelheim, 900 Ridgebury Rd, Ridgefield, CT, 06877, United States; UCB BioPharma SRL, Chemin du Foriest 1, Braine-l'Alleud, 1420, Belgium; Spooner Bioanalytical Solutions, 23 Ashbourne Gardens, Hertford, SG13 8BQ, United Kingdom; ApconiX, Alderley Park, Mereside, Macclesfield, SK10 4TG, United Kingdom; Charles River Laboratories, Elphinstone, Tranent, EH33 2NE, United Kingdom; GSK, 1250 S Collegeville Rd, Collegeville, PA 19426, United States; J&J Innovative Medicine, Turnhoutseweg 30, Beerse, 2340, Belgium; Labcorp, Otley Rd, Harrogate, HG3 1PY, United Kingdom; National Centre for the Replacement, Refinement and Reduction of Animals in Research (NC3Rs), 215 Euston Road, London, NW1 2BE, United Kingdom

**Keywords:** microsampling, ICH S3A, 3Rs, non-rodent, rodent

## Abstract

Adoption of a blood microsampling technique can reduce or avoid the use of satellite animals (rodents) for toxicokinetics or other purposes in discovery and toxicology studies and provides refinements applicable for both rodents and larger animals. Microsampling can increase the scientific value of data obtained from rodent studies during drug and (agro)chemical development, enabling multiple endpoints to be investigated and compared in an individual animal in the same way as non-rodents. A cross-sector survey was developed to understand the current use of microsampling in toxicology studies, with the aim of identifying the specific studies in which microsampling was employed and the barriers to wider uptake. A high proportion of the survey responses indicated that microsampling was used, however, the extent varied widely. Some organisations use the technique only in non-GLP studies. Microsampling was used most for pharmacokinetics or toxicokinetics, commonly within small molecule and agrochemical toxicity studies, but less frequently within large molecule, cell/gene therapies or industrial chemical studies. A wide variety of barriers to wider use of microsampling were provided, typically around reticence to change from using larger samples, or not wishing to validate another bioanalytical method given the resources and challenges associated with the validation of a new technology. Despite these barriers, some organisations have adopted microsampling routinely across many/all rodent toxicity studies and there are opportunities to further reduce and refine animal use across all sectors by wider adoption of microsampling.

## Introduction

**Fig. 1:**
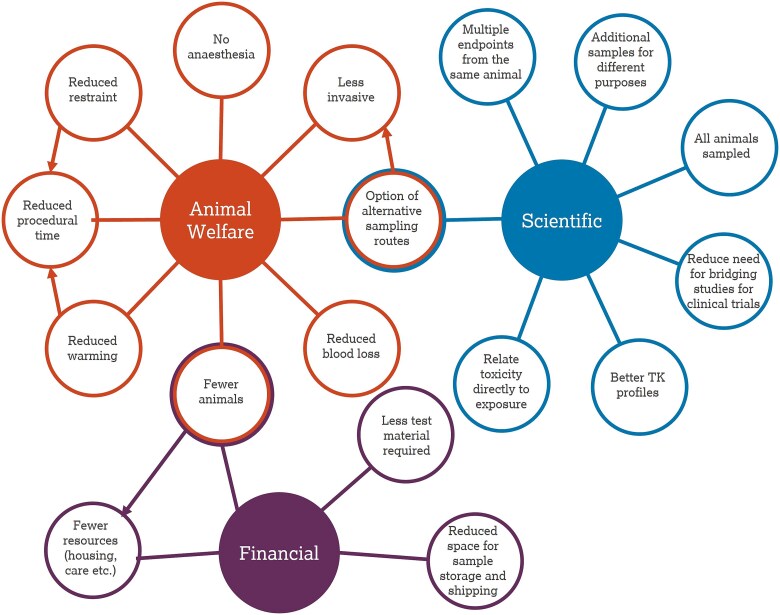
Scientific, animal welfare and business benefits of microsampling in nonclinical studies.

Microsampling is a refined sampling technique for withdrawal of small (typically 25–50 μL) volumes of blood or other bodily fluids (e.g. urine) from laboratory animals. This manuscript focuses on blood microsampling and therefore, throughout the text, the terms “microsampling” and “blood microsampling” will be used interchangeably. Whilst various microsampling methods have been evaluated, including dried blood spots (DBS), dried matrix spots (DMS) and more recently volumetric absorptive microsampling (VAMS), the technique of plasma or capillary microsampling is frequently used for pharmacokinetic (PK) and toxicokinetic (TK) assessments within toxicity studies.^1–5^ Since the introduction of the technique in the mid 2000’s within the pharmaceutical industry via the various methods mentioned above, there has been support for wider application across both investigative and regulatory studies within drug development programmes.^6^ Additionally, the numerous benefits of microsampling (see Fig. 1, and discussed further below) have been recognised and applied within other sectors including agrochemicals.^7^ The benefits of microsampling encompass both scientific and business (financial) drivers, but it is the animal welfare aspects and the potential for both refinement and reduction, two of the “3Rs” (replacement, refinement and reduction), that are arguably the greatest drivers for wider implementation. A summary of the main benefits of microsampling are outlined as follows:

▪ The use of satellite animals for TK purposes is the most common reason for additional animals on rodent toxicology studies, as the small body size and limited blood volumes of rodents restricts the number of samples that can be taken within a specific time period from main study group animals as defined by regional legislation (with oversight by ethical approval committees). The use of smaller sample volumes, facilitated by microsampling, can reduce the number of satellite animals or even remove the need for these satellite groups. This can reduce the number of rodents on a study by more than 40%.^8^ The microsampling procedure itself has multiple benefits including it is quicker, often performed using a more accessible vessel (e.g. tail vein in rodents, or marginal ear vein rather than jugular or cranial vena cava in minipig thus minimal/shorter restrain procedures), involves less blood loss and is deemed to be less stressful to the animal. Additionally, these refinements are applicable for any species and are often incorporated into non-rodent studies in addition to rodent studies.^9^
▪ By microsampling main test rodents, multiple endpoints can be investigated and compared in the same animal, similar to the interpretation of data that routinely happens in non-rodents (e.g. direct correlation of exposure and toxicity data in the same animal). A proportion of the sample can be used for multiple purposes, for example metabolite analysis, biomarkers, genomics, assessment of incurred sample reanalysis (ISR), or stored for future use/repeat analysis if required.
▪ A reduction in the number of animals required for each study can provide cost-savings from supply, housing, husbandry and study procedure perspectives (dosing and sampling of fewer animals), as well as lower test article requirements. Additionally, the smaller samples typically occupy less space in refrigerators/freezers and incur reduced sample shipping costs through smaller packages and reduced dry-ice requirements for example,^10^ although capillary tubes may still need to be stored in larger tubes to allow for proper labelling.
▪ Whilst there has always been a requirement for TK assessments in regulatory toxicity studies for investigational medicinal products, this data was not generated routinely in toxicity studies in other sectors (e.g. agrochemical or chemical industries). Recognition of the potential value of TK data (e.g. to inform top dose setting via an understanding of dose-proportionality or to improve data interpretation), along with the advances in the microsampling techniques and bioanalysis methods developed in the pharmaceutical industry, has led to an increase in the incorporation of TK measurements into agrochemical toxicity studies.^7^^,^^11^
▪ The miniaturisation of nonclinical blood samples has led the way for patient-centric sampling in clinical trials and post-marketing patient populations.^12^ This is particularly useful in paediatric and non-hospital settings and the technologies being used can be adopted for nonclinical purposes. Using the same analytical methodology nonclinically and clinically can reduce the need for bridging studies between different methods or matrices as drug development progresses (another 3Rs benefit).

Various industry consortia and individual companies have advocated the use of microsampling, working together to publish evidence and recommendations for wider implementation within toxicology programmes. Collaborations within the bioanalytical community (e.g. the European Bioanalysis Forum (EBF), and American Association of Pharmaceutical Sciences (AAPS)) have focused on developing the bioanalytical processes and reproducibility of data obtained from microsamples.^13^^,^^14^ The National Centre for the Replacement, Refinement and Reduction of Animals in Research (NC3Rs) has a long-standing working group for microsampling, providing support (data/evidence) to encourage uptake of microsampling and the associated 3Rs benefits to more animals.^15^ Further support for wider implementation of microsampling within drug development was provided via regulatory acceptance of the technique by publication of an International Council for Harmonisation of Technical Requirements for Pharmaceuticals for Human Use (ICH) S3A Q&A document “Note for Guidance on.

Toxicokinetics: The Assessment of Systemic Exposure in Toxicity Studies – Focus on Microsampling.”^16^ Since this time, more companies in more regions have published data and/or have adopted the use of microsampling for different rodent toxicity studies. This includes PK,^17–19^ general toxicity studies,^4^^,^^20–22^ safety pharmacology studies,^23^ developmental and reproductive studies^24–26^ and juvenile toxicity studies.^27^^,^^28^

The current NC3Rs microsampling working group comprises experts in nonclinical toxicity and/or bioanalysis assessments from the UK, Europe and USA, representing 14 pharmaceutical and biotechnology companies, 2 agrochemical companies, 5 contract research organisations (CROs) or consultants and 2 UK academic institutions, and it includes individuals that have worked at regulatory agencies. The aim of this phase of the project was to investigate how widely blood microsampling has been adopted within studies performed for drug and (agro)chemical development (e.g. efficacy, PK, toxicology studies etc) and to gather information on the 3Rs impacts and current concerns or barriers for use of this technique in nonclinical studies. The collated data provides insight into the use of microsampling across different laboratory species, specific studies and drug or chemical modalities, highlighting opportunities for wider application across drug/chemical discovery and development.

## Methods

### Data collection

An Excel-based survey was developed and reviewed by the NC3Rs Microsampling working group. Ethics approval was given by the Social Science Research Ethical Review Board at the Royal Veterinary College, London UK (URN SR2020–0257). The survey was distributed within the working group and more widely via NC3Rs newsletters, other scientific societies or associations and social networking sources (e.g. LinkedIn, X/Twitter etc). Surveys were directed towards those working in industry (e.g. pharmaceutical, agrochemical, consumer products sectors etc), from CROs, or from those in charities or academia running PK or early toxicology/tolerability studies. Responses from those naïve to microsampling technique(s) in their organisations were requested and deemed equally valuable to responses from those currently using, or with previous experience of using microsampling in one or more study types. Completed questionnaires were submitted during the period December 2020 to April 2021 and data were collated, anonymised and analysed by the NC3Rs for discussion with the working group.

The survey consisted of four main sections (see Supplemental Data for a survey outline):

1) Demographic information on the submitting company: including sector, region and molecule type (e.g. drug modality, chemical etc) the organisation was developing.
2) Sampling and study information: including whether microsampling was used for the molecule types being developed, the main and other purposes for the microsamples, the microsampling route for various laboratory species, techniques used, study types generally employing microsampling and the study designs used.
3) Barriers and opportunities for microsampling: including refinements or other scientific benefits identified, approximate reductions in animal use when microsampling was included, or the rationale and barrier(s), if microsampling was not routinely used.
4) Regulatory Acceptance: if any questions on data generated from microsamples had been received from regulatory authorities.

Survey respondents had the opportunity to provide additional information via free-text comment boxes.

### Data analysis

When multiple replies were received from the same organisation (e.g. within one CRO), these were included in the analysis as separate responses when the data reflected different practices and experience at distinct sites worldwide or between different responsible departments (e.g. drug metabolism and pharmacokinetics, DMPK, and safety assessment). The individual survey responses were collated into databases and summarised for the different survey questions. Where appropriate, responses were categorised per sector (pharmaceutical, agrochemical, CRO, academia, other).

## Results

**Fig. 2:**
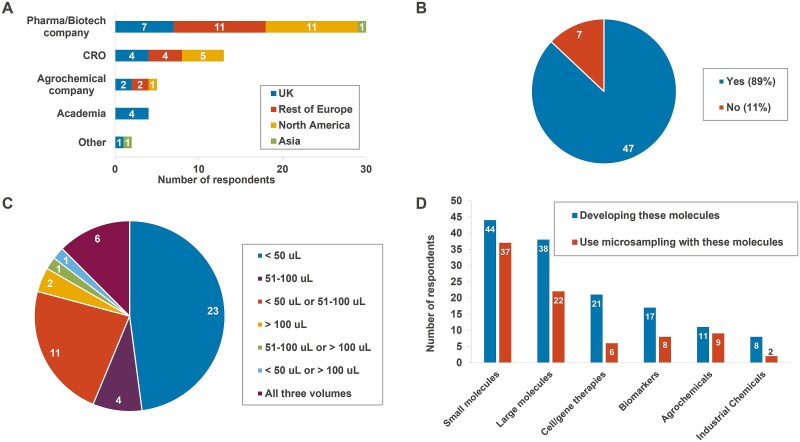
A) Survey respondents by sector and geography. Other* = food company (*n* = 1); government research facility (*n* = 1). B) Survey responses to the question “Have you used microsampling for withdrawal of biological fluids from any animal and study type?”. C) Survey responses to the question “Our microsample volumes are typically: ___”. Survey choices were < 50 μL, 51–100 μL or > 100 μL. Some survey respondents ticked multiple boxes, indicating different microsample sizes per species or purpose. D) Survey respondents working with different molecule types and their use of microsampling with those molecules.

A total of 54 surveys were received (Fig. 2a) from 44 different organisations. Two CROs and four pharmaceutical companies provided separate surveys concerning different sites or departments. Another seven pharmaceutical companies provided a single, consolidated survey with responses on microsampling approaches harmonised across global sites and departments. There were no instances of duplicated surveys from the same company site/department. The surveys originated from respondents in the UK (18), rest of Europe (17), USA (17) and Asia (2, Japan).

Whilst the majority of surveys (89%) were received from companies with experience of microsampling, there were seven organisations who were naïve to microsampling (i.e. microsampling not used) (Fig. 2b). These were three small pharmaceutical/biotechnology companies (two in Europe, one in USA), one large pharmaceutical company (Europe), one full-service CRO (USA), one analytical services CRO (Europe) and one food products company (Asia).

### Microsampling techniques and purpose

The microsampling blood volumes employed varied across organisations and different study types, but 48% of respondents used microsamples under 50 μL and 79% used microsamples under 100 μL (Fig. 2c). The most common techniques were the use of small blood tubes (e.g. Microvette®, KABE) or glass capillaries (e.g. Vitrex®), with direct pipetting, DBS and VAMS used infrequently (supplemental data Table 2e). The main purpose for obtaining the microsamples was predominantly for PK or TK samples for plasma/serum exposure concentration assessments (83% of surveys), with microsampling also being used or trialled for other purposes including clinical pathology samples (blood chemistry and haematology), pharmacodynamic (PD) samples to follow effects over time, biomarkers (efficacy, safety or diagnostic), immunogenicity and DNA/RNA extraction (supplemental data Fig. 2c).

### Microsampling use for different molecule types

Small molecule medicinal products feature most widely in the survey (44 respondents) with microsampling used on 84% of these molecules (Fig. 2d and also see supplemental data Table 1e for a breakdown of different sector responses). Whilst only 11 respondents worked with agrochemicals (five companies and six CRO sites), the majority of these (82%) also used microsampling on these studies. Microsampling was used with less frequency for other medicinal product modalities such as large molecules (58%), advanced therapies (29%), as well as biomarker development (47%) and industrial chemicals (25%), with a general trend of reduced microsampling requested and used across CROs.

### Microsampling use across different nonclinical studies

**Fig. 3:**
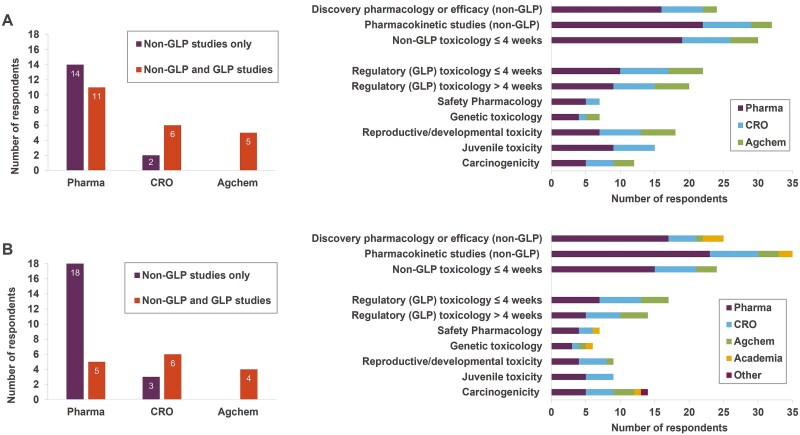
Use of microsampling in different rat (A, top row) and mouse (B, bottom row) studies across discovery and development programmes, by GLP status (left panels) or specific study type (right panels). Other: Government research establishment.

The use of microsampling was clearly more common in early PK, efficacy or investigative toxicology studies in both rats and mice, when compared to later regulatory studies (Figs. 3a and b). The former studies are generally not required to be performed to good laboratory practice (GLP) standards, whereas pivotal (regulatory) toxicology studies are generally required by global regulatory authorities to be performed in compliance with the principles of GLP. The survey shows that some organisations use microsampling only in non-GLP nonclinical studies (e.g. 14 pharmaceutical companies and two CRO sites for rat studies) whilst others use microsampling in a wide variety of non-GLP and GLP studies (e.g. 11 pharmaceutical companies, six CRO sites and five agrochemical companies; Fig. 3a). This finding is also reflected by the highest number of respondents using microsampling in exploratory PK (non-GLP) studies, typically conducted during discovery, and subchronic non-GLP general toxicology studies *<*4 weeks (Fig. 3a right panel). However, microsampling was also being used by some organisations across a variety of pivotal toxicology studies included in the regulatory package, such as GLP general toxicology studies of *<*4 weeks and > 4 weeks duration, reproductive and developmental toxicity, juvenile toxicity and carcinogenicity studies (Fig. 3a right panel). A similar trend is exhibited for studies using mice, with a greater split between 18 pharmaceutical companies using microsampling for non-GLP nonclinical studies only and five using microsampling in a wide variety of non-GLP and GLP studies (Fig. 3b). Some organisations are also using microsampling in the non-GLP and GLP studies in non-rodents (supplemental data Fig. 2f).

When microsampling is included in a toxicity study for PK/TK purposes, sampling of main test animals (either serial sampling at all timepoints, or composite sampling at sparse timepoints) were the designs most regularly employed in mice and rats (supplemental data Table 2g), avoiding the need for TK satellite animals. The survey results suggested that studies involving separate TK satellite groups also occasionally employed microsampling, allowing the use of lower numbers of satellite animals compared with conventional sample volumes.

### 3Rs and other benefits identified

**Fig. 4:**
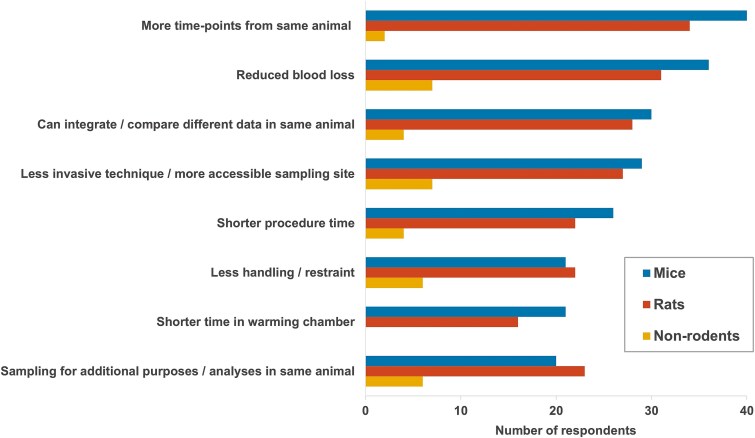
Refinements and other scientific benefits achieved with microsampling.

Survey respondents reported a variety of animal welfare benefits and refinements of microsampling applicable for mice, rats and non-rodents, including reduced blood loss, less invasive technique, or use of a more accessible sampling site, shorter procedure times and less handling/restraint (Fig. 4). Scientific benefits reported included more timepoints from the same animal (potentially providing a more accurate/robust PK/TK profile), the ability to integrate or compare different data within the same animal (e.g. correlation of exposure and toxicology data) and the potential for different sampling purposes/analytes within the same animal. However, it should be noted that the benefits of microsampling in terms of smaller volumes needs to be balanced with animal welfare considerations based on the number of needlesticks (venepunctures), which can become a limiting factor based on restrictions within the licence agreement (e.g. project licence holder in the UK) and ethical review process.

**Fig. 5:**
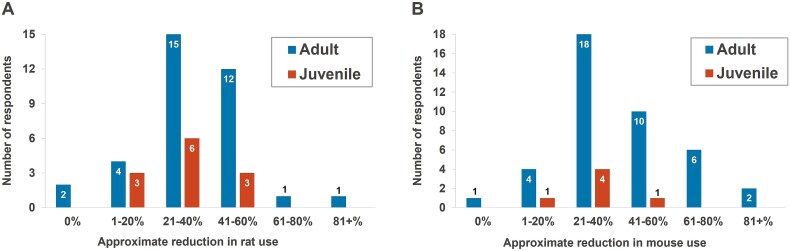
Reductions in animal use achieved with microsampling in rats (A, left) and mice (B, right).

Respondents were asked to estimate an approximate reduction in animal use across their facility when microsampling had been employed in the previous two years (in comparison to previous designs using separate satellite groups of rats/mice; Fig. 5). For adult rats, the most frequently reported reductions were of 21%–40% (43% of respondents) or 41%–60% (34% of respondents), but two respondents indicated a 61%–80% or 81% + reduction in animal use (Fig. 5, left panel). For adult mice, the most frequently reported reductions were of 21–40% (44% of respondents) or 41%–60% (24% of respondents), but six respondents indicated a 61%–80% reduction and another two a ≥ 81% reduction in animal use (Fig. 5, right panel). Where respondents indicated that no reductions in animal use had been achieved, this was accompanied by a comment that blood microsampling was a long-established technique and thus it was difficult to quantify any reduced animal use over the time period specified.

### Reasons and barriers preventing wider use of microsampling

A wide variety of reasons or barriers to wider use of microsampling were provided, along with many additional comments (Table 1 and supplemental data Table 3c). The most frequently cited concerns were that multiple blood samples were needed on a study for different purposes (e.g. ISR, anti-drug antibodies (ADA), TK/Biodistribution, clinical chemistry, biomarkers etc), so larger volumes using established conventional sampling techniques were required (28 responses), that the bioanalytical method was validated for a larger volume of sample and there was a wish to avoid more validation or mixture of methods (21 responses). Other bioanalytical concerns included that blood microsampling had been trialled, but it was not suitable for the molecules being worked with (eight responses) and a lack of conviction that the bioanalytical properties of microsamples (e.g. assay sensitivity, homogeneity etc) were acceptable (eight responses). From a technical perspective, there were responses indicating that the technique was not set-up and there was a lack of experience/trained staff for both sample collection and analysis (eight responses), but also other facilities that are able to perform blood microsampling but were not often requested for this (18 responses, 10 of which were CRO sites). From an in vivo perspective, there were concerns regarding additional samples from main test animals affecting toxicity thresholds (five responses) or clinical pathology profiles (five responses), disturbance to the main test animals or other data being collected (three responses) and the potential additional burden to animals if a higher number of sampling procedures were performed (two responses). Of interest is that concerns regarding regulatory acceptance were only raised by seven respondents and 14 respondents indicated difficulties due to perceived aversions to change away from standard (historical) processes.

**Table 1:** Reasons preventing use of microsampling.

Reasons*	Sector responses					Total
	Pharma	CRO	Agchem	Academia	Other	
Need multiple samples (ISR, ADA, clin chem, biomarkers etc) so need larger volumes	17	9	1		1	28
Bioanalytical method validated for larger samples (avoid validation or mixture of methods)	10	9	1		1	21
We are able to perform microsampling, but are not often requested for this	6	10		2	1	18
Aversion to change away from standard (historical) processes	7	5	1	1		14
Technique not set-up: lack of experience/trained staff (sample analysis)	5	3	1			9
We have tried microsampling but it is not suitable for the molecules we work with	6	2				8
Not convinced the bioanalytical properties are acceptable (assay sensitivity, homogeneity etc)	8					8
Technique not set-up: lack of experience/trained staff (sample collection)	5	2	1			8
Need larger volume samples to perform Thyroid Hormone analysis	2	3	3			8
Concerns regarding regulatory acceptance	4	2			1	7
Concerns regarding additional samples from main test animals affecting toxicity thresholds	4				1	5
Concerns regarding additional samples from main test animals affecting clinical pathology profiles	2	3				5
Difficulty in sourcing or working with tiny tubes/small equipment	3	1				4
Concerns regarding disturbance (to the animals or the other data being collected)	1	1			1	3
Concerns regarding additional burden to animals due to higher number of sampling procedures	2					2
Concerns regarding risk of cross-contamination	1					1

^a^Respondents were able to select multiple reasons for the pre-populated survey answers above, and/or provide free-text comments (these are included in the supplemental data Table 3c).

### Use of microsampling for clinical pathology

**Fig. 6:**
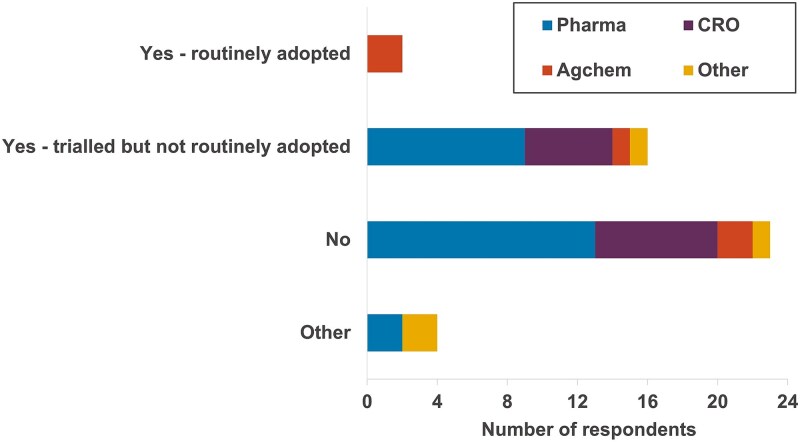
Survey responses to the question “Have you considered using microsampling for clinical pathology sampling?”. Other reasons: don’t include clinical pathology samples in our studies (*n* = 1); not offered by CROs (*n* = 1); not applicable, our clinical pathology samples are terminal bleeds at end of experiment.

A high number of survey respondents (16, including nine pharmaceutical companies and five CRO sites) indicated that they had trialled microsampling for clinical pathology samples, but had not routinely adopted the technique (Fig. 6). A further 23 respondents had not considered using microsampling for these different routine samples. The most frequently cited concerns (Table 2, with additional comments in supplemental data Table 3d) were that the current analytical equipment is intended for clinical samples and require larger volumes of blood for analysis of all standard parameters (33 responses) and that although microsamples could potentially be diluted for use in this equipment, the sample homogeneity was not acceptable (10 responses). Whilst equipment designed for veterinary or paediatric samples (designed for smaller volumes of blood) are available, they are not high-capacity analysers compatible with automated data collection and would therefore need additional validation/qualification for high throughput use, and acceptance by professional organisations and regulators (four responses).

**Table 2:** The concerns or barriers to adoption of microsampling for clinical pathology samples.

Reasons*	Sector responses				Total
	Pharma	CRO	Agchem	Other	
Current analytical equipment requires larger volumes of blood for analysis of all standard parameters	18	10	4	1	33
Homogeneity of microsamples if diluted for use in current standard analytical equipment	3	4	2	1	10
Concerns regarding additional samples from main test animals affecting clinical pathology profiles	5	2			7
Equipment designed for veterinary or paediatric samples (smaller volumes of blood) needs additional validation/qualification before use and acceptance	2	2			4
Not recommended/supported by tox path societies (e.g. STP)		2	1		3
Equipment designed for veterinary or paediatric samples (smaller volumes of blood) is not accepted by regulatory authorities	1				1

^a^Respondents were able to select multiple reasons for the pre-populated survey answers above, and/or provide free-text comments (these are included in supplemental data Table 3d). STP: Society of Toxicologic Pathology.

## Discussion

The purpose of this survey was to gather information on the use, application, 3Rs impacts, barriers and opportunities for blood microsampling within nonclinical studies for pharmaceutical and (agro)chemical products. Whilst the majority of survey respondents were using blood microsampling (either for specific studies or more widely across discovery and regulatory programmes), responses were obtained from some companies where blood microsampling was not implemented. A wide variety of barriers were identified from both those respondents naïve to microsampling as well as those that have implemented blood microsampling, in relation to commonly identified problems when microsampling was introduced in their establishments and/or ongoing problems for specific nonclinical studies.

### Addressing barriers for further adoption of microsampling

In a previous NC3Rs survey,^6^ five main concerns preventing greater uptake of microsampling were identified as follows: 1) sampling may interfere with toxicological end points; 2) sampling sub-groups of animals means that individual animals are being treated differently, which may impact results; 3) practical difficulties with sampling methods; 4) assays may not be sensitive enough from a bioanalytical perspective; 5) concerns over acceptance of the data by regulators. These concerns have largely been addressed over the past decade by the toxicology and bioanalytical communities working together, sharing experience and data to provide reassurance and evidence for the acceptability of microsampling within toxicity studies. However, the current survey data suggests that some concerns around these topics still remain.

#### Regulatory acceptance

The publication of ICH S3A Q&A “Focus on microsampling” relating to medicinal products in 2017 acknowledged the development of the technique and how inclusion within any type of toxicology study within drug discovery and development was acceptable to generate TK data.^16^ Since this time there has been an increase in scientific literature and poster presentations at scientific conferences documenting the validation and implementation of microsampling for TK assessment from individual companies and CROs worldwide. Perceptions around regulatory acceptance were one of the greatest barriers in previous surveys^6^ and would appear to be largely resolved following release of the Q&A document. The data in this survey reflects this, with many companies using blood microsampling in a wide variety of GLP studies within the nonclinical package, with regulatory questions rarely received. However, comments remain around regulatory acceptance, largely from microsampling naïve companies and/or those restricting the technique to non-GLP studies. On occasions, questions from regulatory authorities regarding discordance in datasets have led to bridging studies,^29^ however, this is not a common occurrence and only one survey respondent reported a similar example (see supplementary data 4a/b), as they switched from regular to microsampling within the programme. Encouragingly, a number of respondents reported that microsampling data has been included in regulatory submissions. Furthermore, whilst the ICH S3A Q&A defined a microsample as “typically ≤50 μL”, the survey demonstrated that sample volumes smaller than 100 μL were also commonly defined as microsamples within the community, and still provided an array of scientific and animal welfare benefits.

The majority of the Organisation for Economic Co-operation and Development (OECD) test guidelines for regulatory toxicity studies which are generally used as part of data requirements for agrochemicals or industrial chemicals do not include the requirement for TK assessments or, where this is included, it is optional. For example, in OECD Guidance document no. 116 in relation to chronic and carcinogenicity studies (supporting OECD test guidelines 451, 452 and 453), this is still a flexible endpoint (“*useful information on repeat-dose toxicokinetics may be generated*”) but satellite animals are discouraged (“*a minimum required number of blood samples to calculate representative toxicokinetics can be obtained from the study animals without compromising the outcome of the toxicity study*”). Interestingly the survey data indicates that TK samples are widely included in (agro)chemical toxicity studies, with microsampling used by the majority of respondent companies and CROs working with these chemicals. Although surveys were only received from five agrochemical companies, these were all large multi-site organisations and provide a representation of current activities in this sector. This may reflect an increase in use of refined dosing approaches to provide more relevant data (e.g. dose proportionality assessment etc, see below), for which some knowledge of exposures achieved within study animals is required. The integration of TK into in vivo studies and avoiding the use of additional animals through microsampling, has been acknowledged as a powerful tool to investigate systemic exposures and inform appropriate dose selection.^6^^,^^30^

#### Practical considerations

The adoption of microsampling techniques in scientific research has been facilitated by the increasing availability of specialised equipment and blood collection devices, enabling technicians to obtain small volumes of blood samples efficiently and accurately. Despite its benefits, microsampling is sometimes perceived as challenging, requiring specialised training that may initially take longer than established conventional methods. Sponsors may express caution when considering microsampling techniques, leading to reluctance in requesting them from CROs, despite their availability. This can lead to a difficult situation, as the CRO needs a sufficient influx of microsampling studies to maintain competence of the technical staff performing the sampling.

In GLP studies, slightly larger blood volumes may be necessary to account for repeat analysis, analysis of metabolites, biomarkers, genomics or assessment of ISR. However, in the case of capillary microsampling, the small plasma volume is typically diluted in buffer to allow multiple aliquoting.^1^ When implementing microsampling in TK studies, researchers should consider the potential exacerbation of toxicity signs in main study animals due to repeated blood sampling, with strategies such as composite sampling to minimise the frequency and total volume of sampling. Microsampling techniques offer the advantage of utilising less invasive methods for blood sample collection across various species, enhancing the experience for both animals and technical staff, while also allowing for faster sample collection and practical efficiencies in research workflows. Additionally, the use of smaller gauge needles further refines the technique, minimizing discomfort and tissue damage during blood collection procedures, contributing to the welfare of research animals.^31^

#### Bioanalysis

The EBP/AAPS bioanalysis community has led industry wide collaborations to investigate and provide advice on best practices and processes for handling and analysis of capillary microsamples.^12–14^^,^^32–34^ Despite this, the barriers with the highest response rates were related to bioanalysis issues. The greatest challenge hindering the adoption of blood microsampling relates to the need for larger sample volumes to allow running of different assays aside from bioanalysis for PK/TK for different endpoint measurements. For example, nonclinical studies often require the analysis of multiple endpoints, including ISR, ADA, TK/Biodistribution, clinical chemistry and biomarkers that can be achieved in a single blood draw (then sub aliquoting) using conventional sampling. Blood microsampling techniques typically provide limited sample volumes, which may prove insufficient to accommodate the diverse analytical requirements across these endpoints. Consequently, some responders face challenges in obtaining the necessary sample volumes for comprehensive endpoint analysis using microsampling techniques. This is specifically perceived as an issue when running toxicity studies with monoclonal antibodies or larger molecular weight constructs that may generate an immune response and require ADA assessment.

The second highest barrier is the reluctance to move away from a validated method using conventional sample volumes. Established bioanalytical methods may have been validated for larger sample volumes obtained through conventional sampling approaches. As a result, some responders exhibit reluctance to deviate from these validated methods, fearing potential disruptions to methodological consistency, reliability and regulatory compliance. This is also driven partly by concerns about the additional time, resources/cost and training required to validate and implement microsampling techniques. There were also indications that some companies were still not convinced the bioanalytical properties for microsamples are acceptable (e.g. assay sensitivity and homogeneity). In terms of sensitivity, this is usually an assay dependent concern which will vary by analyte and instrumentation available to each group or company. Since doses in toxicity studies are relatively higher than clinically relevant doses, these assays do not necessarily require high assay sensitivity, although in the case of some chemicals or routes such as dermal application where there are often low exposures, a low lower limit of quantification is needed in the bioanalytical method to sufficiently characterise the exposure profile. Homogeneity has been flagged as a concern by the bioanalytical community previously, notably in the case of DBS.^35^^,^^36^ This is an ongoing area of investigation that has produced positive results and where the opportunities/benefits are becoming more apparent.^37^ Several large organisations are routinely using microsampling approaches in this space, indicating that these issues are not insurmountable.^9^^,^^38^

#### Other observations

The survey findings highlight a notable trend in exploratory PK studies, wherein blood microsampling techniques are frequently employed for sample collection and analysis. However, despite the effectiveness of these methods in initial PK assessments, they typically use research grade methodology and thus were not validated for subsequent use on regulatory studies, where blood is drawn for multiple endpoints (i.e. clinical pathology, immunogenicity and other biomarkers). Furthermore, this discrepancy may stem from differences in study populations, as early PK studies primarily involve rodents, while regulatory studies also encompass non-rodent species where blood volume constraints are less pronounced. Additionally, there may be organisational factors at play, such as different teams overseeing early PK studies conducted during discovery versus those responsible for pivotal toxicology studies conducted by safety assessment, requiring regulatory compliance. The data indicates that adoption is greatest for PK/TK sampling to characterise exposure, particularly in rodent PK/PD and non-regulatory toxicology purposes. Whilst there are a number of organisations with experience of microsampling in support of regulatory toxicology studies, the utility is nevertheless less prevalent especially in studies involving non-rodent species, indicating a need for further exploration and validation in these contexts. Examples exist in the literature that demonstrate collection of samples with multiple detection endpoints for TK/PK studies have little toxicological influence on study parameters, though these primarily relate to studies in rat.^39–41^

Blood volume requirements can be guided by study design and overall project requirements. Where microsampling has been successfully implemented over multiple studies within drug and agrochemical companies, there has been close involvement between bioanalysis and toxicology teams and the initial “non-GLP” microsampling method development is the one formally validated for GLP studies (one method developed per species). There are likely opportunities to employ microsampling for any new substances in development, where there are fewer concerns around changing methods for well-established programmes.

### Further opportunities for adoption of microsampling

The survey data indicated the following opportunities for wider adoption of microsampling, encompassing the following topics/themes.

#### GLP studies and species

Similar to data published previously,^33^ the greatest use of microsampling was within non-GLP studies, for PK profiling and TK within early investigative pharmacodynamic or toxicology studies. Whilst some companies have adopted microsampling within some or all studies within the GLP regulatory package for pharmaceuticals and agrochemicals, this is an obvious opportunity for wider adoption of the technique, especially given that regulatory acceptance of this data has been in place since 2017. Companies that are using microsampling have reported multiple refinements that improve animal welfare for both rodents and non-rodents. For studies historically employing satellite groups for TK purposes such as carcinogenicity studies, large reductions in animal use can be achieved if microsampling of main test animals is employed.^42^ For other studies, such as reproductive toxicity and juvenile toxicity studies where collection of even one TK sample from extremely young animals is usually a terminal procedure due to limited blood volumes and practicality of collection, microsampling can be of great benefit as even taking just two TK samples from the same animal would half the number of animals required. Microsampling methods are therefore developed for these special studies so there is presumably scope to apply to other regulatory studies such as dose range finding toxicity studies, GLP studies, and smaller non-rodent studies such as non-human primates (NHPs) and rabbits.

Use of microsampling in larger species (e.g. non-rodents) does not provide opportunities for reduction in animal use, since these species generally have sufficient blood volumes to meet requirements for blood sampling well within daily or monthly limits, and satellite animals are not necessary. However, the refinement opportunities that microsampling provides for non-rodents are equally relevant and animal welfare can be improved via less handling or restraint and less invasive techniques. Examples of refinements related to specific species include:

▪ Rabbits: blood samples are generally taken from the marginal ear vein of the rabbit and there is less trauma/quicker recovery using microsampling compared with conventional sampling.
▪ Minipigs: conventional sampling in the minipig is generally achieved from the jugular vein with the animal held in the supine position for ease of access. This can be met with some resistance and particular care may be required to avoid biting incidents. The use of a sling to hold animals in a more natural position and the use of microsampling to collect blood from peripheral sampling sites such as the saphenous and the ear vein greatly facilitates the sampling causing less stress to the animals and reduces the risk of trauma to the animal through reduced need for restraint.
▪ Dog: similar concept to minipigs with regards to using less invasive sampling sites (cephalic/saphenous). Microsampling also allows samples to be collected more quickly, allowing the animal to return to the home pen more quickly, and/or animals could be trained to take samples from within the home pen.
▪ NHPs: cage-side sampling can be employed (whereby the animal is trained to offer a forelimb through the home cage bars) rather than chair restraint; the former method is quicker and less stressful.^43^ Microsampling also avoids the rare situation of the need to anaesthetise animals when larger blood collection samples are required (e.g. vacutainer).

For all species, refinements can include the use of smaller gauges of needle so the animals experience of the sampling procedure is reduced, as well as welfare advantages by reducing the amount of trauma induced from multiple needle sticks (compared with conventional methods). However, microsampling can lead to the risk/temptation for increased number of samples per animal and the benefits of microsampling needs to be balanced with the number of needlesticks (i.e. just because the blood volumes allow more samples, doesn’t mean more should be taken – six timepoints should still be sufficient for analysis of a 24 h profile). However, where there is an increase in the number of TK samples in safety pharmacology non-rodent telemetry studies, for example for fuller profiles and exposure-response modelling adoption of microsampling could still be a refinement.^44^

#### Drug modalities other than small molecules

The application of microsampling has mainly been within studies for small molecules, since these were the prevalent molecule type in portfolios at the time of development of the technique.^45^ More recently, a wider range of modalities are in development, but the use of microsampling in these newer drugs is far below that of small molecules. Why might this be? For large molecules such as monoclonal antibodies (mAbs), often only a single pharmacologically relevant species (generally NHPs) is used in toxicity studies and a conventional larger sample volume can be used. However, with the need for multiple samples for different purposes (e.g. TK, ADA, biomarkers etc) and availability issues leading to use of smaller animals with lower blood volumes, use of microsampling can maintain the required number of samples whilst allowing the refinements mentioned previously to be applied. The analysis methods used for large molecules and other drug modalities differ from those employed for small molecules, with techniques such as ligand binding assay (LBA), polymerase chain reaction (PCR), hybridisation-ELISA (enzyme-linked immunosorbent assay) and liquid chromatography–mass spectrometry (LC–MS) with fluorescence detection being the most common. These methods often require technical replicates, which historically increased sample volume needs compared to the single replicate analysis typically employed in LC–MS for small molecules. However, this does not preclude the validation of microsampling techniques for large molecules, as evidenced by numerous publications utilising microsampling for mAbs (Caron et al.,^9^^,^^38^^,^^43^) and oligonucleotides.^46^^,^^47^ Notably, singlet analysis, explored by groups such as EBF^48^ endorsed by the ICH M10 guidance, advocates for single replicate analysis, potentially influencing volume demands for other modalities and technologies. This endorsement may pave the way for further adoption of microsampling in preclinical studies involving large molecules and other drug modalities.

#### Agrochemicals

The aforementioned benefits of microsampling have also been utilised within agrochemical research up to and including data that was incorporated into regulatory dossiers. Traditionally TK sampling was not included in toxicity studies with agrochemicals but is increasingly included to link exposure with findings and the use of microsampling avoids the use of satellite animals. In practical terms, sample volume limitations can be minimised by addition of water to the blood samples e.g. an equal volume of water added to each 50 μL blood sample. Following sample extraction for bioanalysis, remaining volumes from the sample supernatants can be pooled in order to permit subsequent analysis for metabolites. Furthermore, with the low volume of blood required for TK profiling, this permits further blood aliquots from the same venepuncture sampling occasion to be utilised for high quality data generation such as metabolomics.

The combination of TK and metabolomics profiling from the same animals permits detailed analysis to assess the influence of exposure on endogenous biomarkers of toxicological endpoints. In addition, the full profiles generated from these sampling regimes can add weight of evidence to dose setting for subsequent studies in the cases of sub-proportional exposure across the full dose range i.e. limiting the maximum dose when evidence indicates there was no increase in exposure (e.g. area under the curve, AUC and maximum serum concentration, C_max_) as dose levels increased.

#### Academia

In the small number of UK research establishments that responded to the survey, microsampling appears to be well established for TK, biomarker and pharmacodynamic response purposes. The low response rate may perhaps be due to different terminologies, since it is known that small volumes of blood are often used for monitoring pharmacodynamic profiles such as blood glucose measurements, but as the small whole blood sample differs from the capillary microsamples used within industry these may not be called microsamples. Another reason could also be that microsampling is not widely used due to a lack of experience in the technique and lack of access to training opportunities. Whilst small pharmaceutical/biotech companies can access advice or view the technique at CROs that may be performing other studies for the company, academic researchers rarely have these contacts and often can only rely on previous experience of individuals in the research group or facility. There may be an important role here for the Animal Welfare and Ethical Review Body (AWERBs) and/or the Institutional Animal Care and Use Committee (IACUC) that oversee the animal work of multiple groups within a facility, to encourage sharing of knowledge/expertise across research groups and to establish links with other (external) academic groups to facilitate transfer of practices and procedures. Some organisations provide specific training for regulated procedures, but the working group members are not aware of a practical microsampling course that is openly available.

#### Clinical pathology

Another major purpose of blood sampling within toxicity studies is for clinical pathology purposes (clinical chemistry, coagulation and haematology assessments). The question therefore remains as to whether microsampling can be applied to these samples too, as a refinement in blood volume collections. This was previously investigated by members of the American Society for Veterinary Clinical Pathology (ASVCP)^49^ with recommendations that microsampling was not suitable at that time, due to current analyser (higher) volume requirements and quality assurance requirements. Nevertheless, in the intervening years since this paper many survey respondents had trialled microsampling for clinical pathology purposes, but none had yet implemented this routinely. The main barriers cited for this were similar to those in the previous ASVCP survey, with the majority of respondents stating that current analytical equipment requires larger volumes of blood for analysis of all standard parameters. That said, in recent years, the technology for haematology analysers to measure microsamples has vastly improved with the launch of new haematology analysers. For example, one haematology analyser only require 25 μL of whole blood and can measure up to 60 samples within an hour. The only drawback for this analyser is that it does not offer the profile/configurations for veterinary/lab animal species. An alternative analyser offers the veterinary configurations and only requires 100 μL of whole blood which is still better than other major high throughput haematology analysers on the market. Hence, with increased demand for microsampling in laboratory animals, we hope the manufacturers will include the veterinary configurations in future analysers.

For clinical chemistry, although there are some point-of-care analysers in the market, these analysers are only limited to measuring fewer analytes at a low throughput capacity with no veterinary configurations, which may not be practical for toxicology studies that usually have large number of samples. The current clinical chemistry analysers with high throughput capacity have high dead space volume requirements (50 μL) on top of the additional volume required for each parameter to be analysed, thereby making microsamples impossible for these analysers. To mitigate this issue, pre-analytical dilution of microsamples has been suggested. However, this approach is not encouraged due to concerns over sample homogeneity, haemolysis, assay bias and imprecision, and values falling below assay range which precludes evaluation of bias.^50^ Nevertheless, in some scenarios, clinical chemistry analysis can still be performed with reduced serum/plasma samples without sample dilution by only analysing selected parameters based on the knowledge of the program. This approach can only be taken during preliminary toxicology studies, as the full clinical chemistry panel will be needed for the GLP studies. As the demand for microsampling grows in the veterinary space and the technologies evolve, we hope the diagnostic device manufacturers will consider the development of high throughput clinical chemistry analysers suitable for microsamples.

Given the relatively larger sample volumes for clinical pathology samples (haematology, clinical chemistry and coagulation), there are still unrealised benefits to be gained by developing smaller volume sampling for clinical pathology endpoints in the nonclinical space, even if they would not fall into the “traditional” microsample definition. Given the entirety of the potential endpoints using blood sampling on a nonclinical toxicity study such as TK, clinical pathology, cytokines, target engagement, T-Dependent Antibody Response (TDAR) and/or ADA analysis, blood volume limits can be exceeded and is obviously true for rodents like mice and rats but can also be true for smaller non-rodent species like NHPs and rabbits even if using microsampling approaches for TK analysis. Therefore, the entire community of scientists contributing to the multitude of endpoints on a nonclinical study should continue to work together and share learnings to help broaden the application of microsampling to clinical pathology samples and other endpoints.

## Concluding remarks

Despite the numerous animal welfare (3Rs), scientific and business advantages that adoption of microsampling can provide, barriers still remain within many organisations that restrict microsampling use to non-GLP studies or have not yet adopted the technique at all. Much of this may be due to reticence to change from using larger samples and established practices, the use of new(er) drug modalities where the use of microsampling has not yet been widely demonstrated or those using predominantly non-rodent species where blood volume limitations are not perceived to be problematic or trying to avoid the challenges that come with validations of more sensitive bioanalytical methods.

How can these current barriers be overcome? Some potential approaches include: push-back and oversight from AWERB/IACUC when initial protocols are reviewed, with microsampling encouraged; availability of specific training and/or advice for those wishing to adopt microsampling; close collaborations between toxicology and bioanalysis teams within companies with support from higher management for microsampling; CROs (where the majority of regulatory studies are currently performed) offering the technique as default (for rodents and non-rodents) and only accepting use of rodent satellite groups when absolutely necessary; promotion of the technique during regulatory feedback meetings and commented on when submissions received that used conventional sampling and satellite groups; continued sharing of experience and benefits of microsampling from CROs and pharmaceutical/agrochemical companies. The companies regularly using microsampling have adopted the technique from the beginning of a project, using this during investigative and non-GLP studies such as PK early in development then validating the bioanalysis method for use in later regulatory studies. This is the key mind-set required for wider adoption of microsampling within the pharmaceutical and agrochemical industries. Overall, instead of retaining the method for larger (conventional) volumes and using this for both non-rodents and rodents, if the initial method was a small volume microsampling method suitable for rodents but then adopted for non-rodent studies in addition, this would bring practical advantages (i.e. having a single bioanalysis method for application across multiple studies and species) plus extend the animal welfare benefits of microsampling to non-rodents. Furthermore, exploration of new opportunities for microsampling, such as for other common samples within toxicology studies (e.g. clinical pathology, biomarkers etc) would provide additional refinements to the studies and/or further reductions in satellite animal use.

## Supplementary Material

Microsampling_supplemental_data_Final_Version_tfaf045
