# Supplementary material for: Microsampling in toxicology studies – maximising the scientific, business and 3Rs advantages
Source: Toxicol Res (Camb). 2025 Mar 31;14(2):tfaf045. doi: 10.1093/toxres/tfaf045 (PMC11957253; doi:10.1093/toxres/tfaf045)
Supplement: Microsampling_supplemental_data_Final_Version_tfaf045 [file microsampling_supplemental_data_final_version_tfaf045.docx]

**Excel-based survey (data returned to NC3Rs via email December 2020 to April 2021)**

**Additional results**

1e. Which types of molecules do you currently work with? and 2b. for studies with which molecule types do you routinely include microsampling?

| **Small molecules** | | | |
| --- | --- | --- | --- |
|  | Working with these molecules | Use microsampling with these molecules | % using microsampling |
| Pharma | 27 | 24 | 89 |
| CRO | 13 | 10 | 77 |
| Academia | 4 | 3 | 75 |
|  |  |  | **Overall 84%** |
| **Large molecules** | | | |
| Pharma | 24 | 17 | 71 |
| CRO | 11 | 4 | 36 |
| Academia | 2 | 1 | 50 |
| Other | 1 | 0 | 0 |
|  |  |  | **Overall 58%** |
| **Cell/gene therapies** | | | |
| Pharma | 11 | 2 | 18 |
| CRO | 7 | 2 | 29 |
| Academia | 3 | 2 | 67 |
|  |  |  | **Overall 29%** |
| **Agrochemicals** | | | |
| Agchem | 5 | 5 | 100 |
| CRO | 6 | 4 | 67 |
|  |  |  | **Overall 82%** |
| **Industrial chemicals** | | | |
| CRO | 7 | 2 | 29 |
| Other | 1 | 0 | 0 |
|  |  |  | **Overall 25%** |
| **Biomarkers** |  |  |  |
| Pharma | 6 | 4 | 67 |
| CRO | 8 | 1 | 13 |
| Academia | 2 | 2 | 100 |
| Other | 1 | 1 | 100 |
|  |  |  | **Overall 47%** |
| **Other chemicals, drugs (GMOs)** | |  |  |
| Pharma | 4^a^ | 1^b^ | 25 |
| CRO | 1 | 1 | 100 |
|  |  |  | **Overall 20%** |
| **Other products** | |  |  |
| CRO | 1**^c^** | 0 | 0 |
| Academia | 4^d^ | 1 | 25 |
| Other | 4^e^ | 0 | 0 |
|  |  |  | **Overall 11%** |

a = Oligonucloetide (2), Antibody Drug-Conjugate (1), Peptide (1). b = Peptide. c = Food industry chemicals/products. d = Standard refs/reagents. e = Food industry chemicals/products (1), Particulates (1), e-cigarettes (1), biological allergens (1).

2a. Have you used microsampling for withdrawal of biological fluids from any animal and study type?


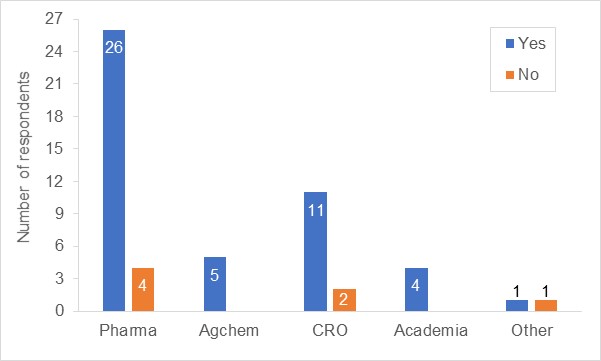


2c. What is the main purpose for which you use microsampling most regularly?


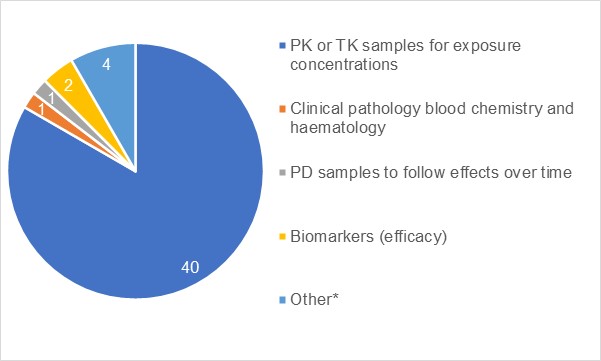

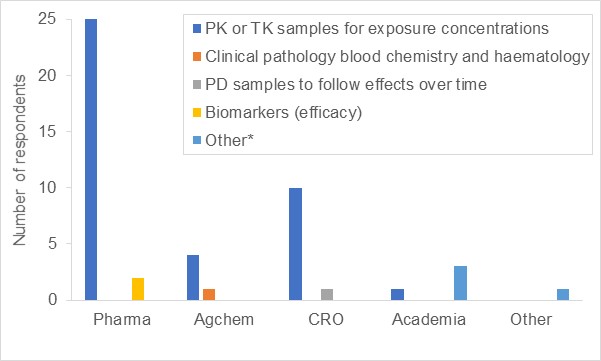


Other* = PK or PD non tox purposes (1); Various (1), CTD detection (1); biomarkers of radiation (1).

And are there other purposes for which you use, or have tried, microsampling?


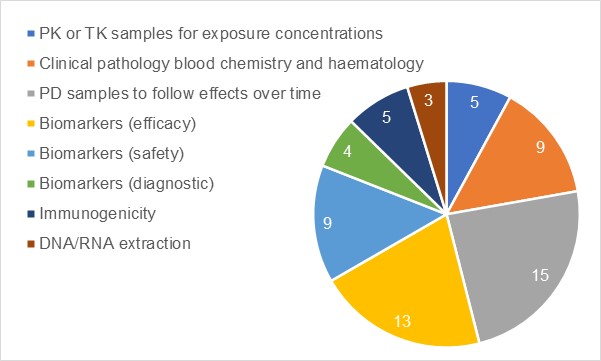


2d. What is the main route you use for microsampling in each species at your facility?

| **Route** | Rat (adult) | Rat (juvenile) | Mouse (adult) | Mouse (juvenile) | Rabbit | Dog | Minipig | NHP* |
| --- | --- | --- | --- | --- | --- | --- | --- | --- |
| Tail vein | 26 | 10 | 23 | 4 |  | 1 | 1 | 2 |
| Tail nick | 4 | 2 | 8 | 6 |  |  |  |  |
| Saphenous vein | 2 | 1 | 7 |  |  |  | 1 | 1 |
| Cephalic vein |  |  |  |  |  | 5 |  | 4 |
| Jugular vein (sedated) | 1 | 1 |  |  |  |  |  |  |
| Jugular vein (conscious) | 3 | 4 | 2 | 1 |  | 6 | 1 |  |
| Submandibular vein (sedated) | 1 |  | 1 |  |  |  |  |  |
| Submandibular vein (conscious) |  |  | 3 |  |  |  |  |  |
| Marginal ear artery or vein |  |  |  |  | 14 | 1 | 8 |  |
| Other route (please specify) |  |  | 2^a^ |  |  |  |  | 1^b^ |

a = vena facialis; orbital sinus; b = femoral; * cynomolgus macaque.

2e. Which microsampling techniques do you use most regularly?

| **Technique** | **Number of responses** |
| --- | --- |
| Smaller blood tube (e.g. Microvette, KABE) | 29 |
| Glass capillary (e.g. Vitrex) | 28 |
| Direct pipetting | 7 |
| Dried blood spot (DBS) | 5 |
| Glass capillary with thixotropic gel (e.g. Drummond) | 3 |
| Volumetric Absorptive Microsampling (VAMs, e.g. Mitra) | 2 |
| Other (Minivette) | 2 |

Which ONE microsampling technique do you use most often?

| **Technique** | **Number of responses** | | |
| --- | --- | --- | --- |
|  | Rat | Mouse | Non-rodent |
| Smaller blood tube (e.g. Microvette, KABE) | 15 | 14 | 7 |
| Glass capillary (e.g. Vitrex) | 15 | 24 | 5 |
| Direct pipetting | 3 | 3 | 1 |
| Dried blood spot (DBS) | 1 | 1 | 0 |
| Glass capillary with thixotropic gel (e.g. Drummond) | 1 | 0 | 0 |
| Volumetric Absorptive Microsampling (VAMs, e.g. Mitra) | 1 | 1 | 1 |
| Other (Minivette) | 1 | 1 | 2 |

2f. For which study types is microsampling regularly included (non-rodents)?

2g. When microsampling is included, which sampling design is most regularly employed?

| **Sampling design** | **MICE** | **RATS** |
| --- | --- | --- |
| Serial sampling main test animals (all timepoints) | 100% n=11 60%* n=21 | 100% n=10 54%* n=20 |
| Composite sampling main test animals (sparse timepoints) | 100% n=2 29%* n=22 | 100% n=1 33%* n=16 |
| Serial sampling satellite animals (all timepoints) | 100% n=1 33%* n=10 | 100% n=3 42%* n=10 |
| Composite sampling satellite animals (sparse timepoints) | 100% n=3 31%* n=11 | 100% n=1 23%* n=9 |

*Mean value for respondents that sometimes use this sampling design

3b. Please pick the TOP THREE study types where the greatest reduction in animals has been achieved at your facility.


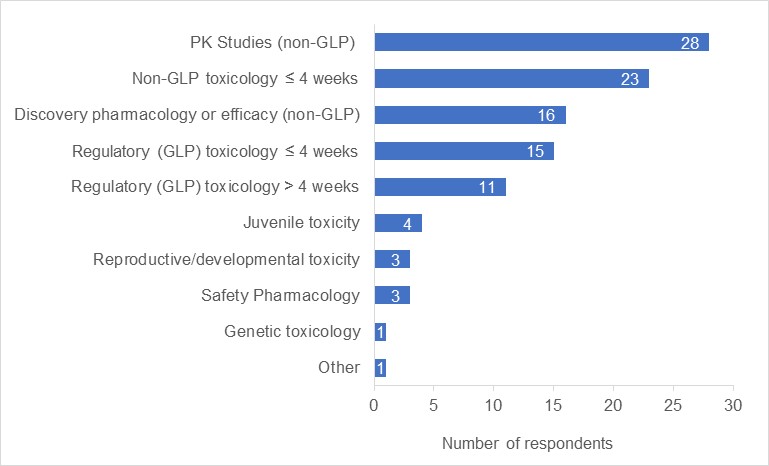


3c. Where microsampling is *not* routinely used, respondents were asked select all the reasons preventing the use of microsampling (Table 1, main manuscript). Respondents were then asked to pick the TOP THREE reasons preventing the use of microsampling (below), with the option to provide additional free-text answers.

| Reasons* | Sector responses | | | | | Total |
| --- | --- | --- | --- | --- | --- | --- |
|  | Pharma | CRO | Agchem | Academia | Other |  |
| Need multiple samples (ISR, ADA, clin chem, biomarkers etc) so need larger volumes | 14 | 6 | 1 |  |  | 21 |
| Bioanalytical method validated for larger samples (avoid validation or mixture of methods) | 6 | 7 |  |  | 1 | 14 |
| We are able to perform microsampling, but are not often requested for this | 1 | 9 |  | 1 |  | 11 |
| Aversion to change away from standard (historical) processes | 4 | 3 | 1 |  |  | 8 |
| We have tried microsampling but it is not suitable for the molecules we work with | 5 | 2 |  |  |  | 7 |
| Technique not set-up: lack of experience/trained staff (sample collection) | 4 | 2 |  |  |  | 6 |
| Technique not set-up: lack of experience/trained staff (sample analysis) | 2 | 2 | 1 |  |  | 5 |
| Concerns regarding additional samples from main test animals affecting toxicity thresholds | 3 |  |  |  | 1 | 4 |
| Not convinced the bioanalytical properties are acceptable (assay sensitivity, homogeneity etc) | 4 |  |  |  |  | 4 |
| Concerns regarding regulatory acceptance | 2 | 1 |  |  |  | 3 |
| Need larger volume samples to perform Thyroid Hormone analysis | 1 |  | 2 |  |  | 3 |
| Concerns regarding additional samples from main test animals affecting clinical pathology profiles | 2 | 1 |  |  |  | 3 |
| Concerns regarding disturbance (to the animals or the other data being collected) | 1 |  |  |  | 1 | 2 |
| Concerns regarding risk of cross-contamination | 1 |  |  |  |  | 1 |
| CRO not amenable to using main study animals for TK sampling | 1 |  |  |  |  | 1 |
| Difficulty in sourcing or working with tiny tubes/small equipment | 1 |  |  |  |  | 1 |
| Concerns regarding additional burden to animals due to higher number of sampling procedures | 1 |  |  |  |  | 1 |
| Client agreement/acceptance |  | 1 |  |  |  | 1 |

Free text answers:

The time it takes for collection, number of people and supplies are FAR more than with other methods.

The main barrier, as a CRO, is gaining client agreement/acceptance to use the technique. This is particularly difficult for non-rodent studies as there is no reduction in animal numbers.

Microsampling is technically challenging, require proper training, implementation can be associated with high incidence of sample collection issues (insufficient volume collected, excessive sample heamolysis, etc). Issue with labeling microcapillary/can lead to errors, need a good labeling system. Handling of the samples more laborious, involved more work for bioanalytical analysis. Should be evaluated for each study the need of microsampling. Risks/cost benefit approach, does not justify in every case especially for GLP tox study.

Not used when there is no scientific benefit or when we can not reduce the number of animals.

For studies contracted out (non-regulatory pilot/DRF and GLP tox studies), we frequently are taking larger sample volumes as we analyse multiple parameters at each timepoint (e.g. TK, biomarker (safety/efficacy) and clin path) to establish a detailed safety and PD profile. To reduce number of sticks, we routinely take a larger volume and then split the sample for relevant analytes. Frequently the PK/TK and biomarker analytes are conducted using microsample volumes (<50 uL blood).

Additional validations if used in GLP studies.

Need larger volume for fluoride analysis.

We have tried microsampling in our laboratory. When some small molecules were administrated to rats, their PK parameters were varied by the difference of procedure (the microsampling from tail vein and the conventional sampling from jugular vein). In addition, we could not collect blood from the tail vein of several animals within the time defined in Standard Operating Procedures.

CRO not amenable to using main study animals for TK sampling.

Often we do not need to take samples during the study - we just collect the whole blood at the end when the animal is killed. We also analyse a few different metabolites and so possibly may need greater volume to do this.

Generally, the majority of our clients and bioanalytical sites/CROs try now to validate the method with microsampling instead of larger volume but it's not always possible depending on the type of analysis/molecule. In addition, we propose microsampling to the client from the 1st study in a project.

We have performed test studies to confirm DBS and VAM's work in our hands, but there has been no demand from our clients.

We tried microsampling, and not suitable in terms of little benefit *vs* high risks in support of GLP tox studies.

Microsampling is used routinely, but training staff to collect and process samples consistently and reliably is a concern.

Usually for biologics, multiple analytes need to be analyzed from the same sample so larger sample volumes will be required. And LBA assays are usually employed for biologics which will need larger volume for accurate sample dilution.

Validated an acceptable liquid microsampling method in NHPs for large molecules, however, bioanalytical personnel do not want to do the additional work to validate methods for small molecule programs.

3d. What are the concerns or barriers to adoption of microsampling for clinical pathology samples?

Free text answers:

Currently the CROs we use are not able to offer microsampling and dilution of blood for the haematology and biochemistry parameters we use. We would be interested in using microsampling if we were certain that all parameters would be measured accurately in a GLP compliant way. We are waiting for more peer reviewed papers to indicate that micosampling and dilution of blood give consistently reliable clinical pathology results.

Most current clinical analyzers use low volume sampling. For all of the guideline/protocol required clinical chemistry parameters, 150uL of serum is needed. In some situations (clinical chemistry), samples are diluted prior to analysis so that more parameters can be measured. Before initiating diluting serum samples, testing of undiluted and diluted samples is performed to verify reporting results from diluted samples is acceptable.

It has not been an accepted practice to dilute samples to obtain enough quantity to analyze standard parameters. Once accepted everything would require a method validation to ensure biological integrity of results.

It is absolute possible, but today there is no need, in our studies we always take blood samples for clinical pathology during anaesthesia just before necropsy. Our analytical equipment (Vetscan) needs 100µL whole blood/animal and our analytical equipment for running haematology samples needs 20µL whole blood/animal.

All historical data is based on standard sampling.

We have no experience.

Microsampling for Clinical pathology has been considered in the UK but not yet implemented. Clinical pathology samples for non-GLP rodent studies are terminal samples and the maximum volume is collected to ensure adequate sample available for repeats or additional endpoints (immunotox, pharmacodynamic, hormone analysis, etc), so no advantage to use microsampling.

Not sure of regulatory acceptance.

Routine clin path samples only taken on non-regulatory and GLP toxicology studies. CROs are reluctant to reduce sample volumes for haematology, clinical chemistry and reasons that have been cited include technical limitations (equipment & analytical headspace) and desire to have larger sample for possible repeat analysis (e.g. in case of out-of-range data etc). The risk of this is, at least anecdotally, low in our opinion and benefits of reduced sample volumes would generally outweigh risk of possibly losing the odd data point from an animal - or even a complete sample profile as we are typically sampling 5 or 10 animals per group/sex and 1/5 or 1/10 wouldn't compromise the integrity of the study in our opinion.

Need appropriate collection procedure/equipment and bioanalytical methods for microsampling; regulatory consideration is a concern as well.

Well established use and application of microsampling from bioanalytical purposes. There is appetite and need for microsampling for traditional haematology and clinical chemistry analysis - but analytical capability is limited/not well validated at this time. Efforts to explore this have occurred at some sites for example with mouse haematology since blood volume even at termination is limited. Often this means additional animals are added to studies to accommodate all the sample requirements. Would consider microsampling for clinical pathology samples, however, none of the CROs we currently work with have offered this option with an evidence base that it provides all the parameters required on a toxicology study.

4a. Have you experienced any questions from regulatory authorities regarding microsampling or the PK/TK data generated?


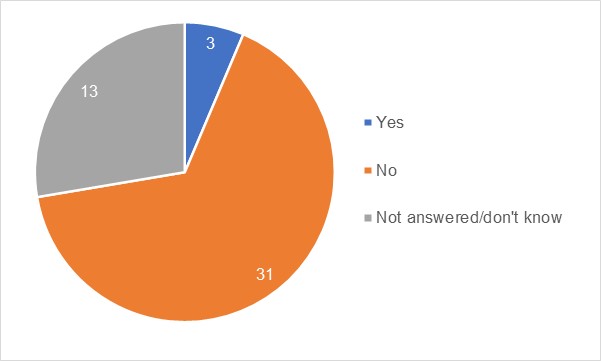


Comments for the ‘Yes’ responses: 1) From Home Office Inspector, regarding how the study was designed in order to have enough sampled without comprising the PPL limits; 2) We were asked by FDA to perform a bridging study between regular plasma sampling and plasma microsampling because we switched from regular to microsampling within the program; 3) Questions were focused on the validation parameters evaluated and bridging to plasma concentration data.

4b. Do you regularly perform bridging studies (using additional animals) due to requests for additional information or other concerns?


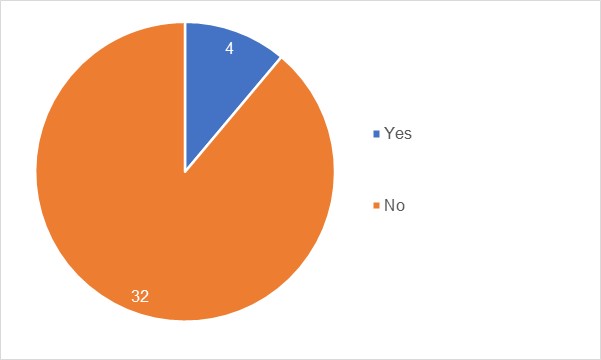


Comments for the ‘Yes’ responses: 1) usually for formulation changes; 2) in cases where we observe significantly variability in PK between studies; 3) when switching matrix (e.g. from plasma to DBS).

4c. For approximately how many test compounds do you have PK/TK profiles from conventional sampling and microsampling?

Eleven responses as follows: One compound (2 respondents); 3 to 4 compounds (1); 4 compounds (1); less than 5 compounds (1); at least 5 compounds (1); 6 test compounds (4 small molecules, 1 peptide, and 1 large molecule) which are commercially available (1); ~15 compounds (1); ~5 development programs; 80% discovery programs (1); about 100 compounds (1); > 5,000 (1).

4d. For how many of these (%) are the PK/TK profiles in concordance? Has this data been published?

Ten responses as follows: all of them and no, not published, just presented in a poster at a conference; 100% most of the data has been published; 4 compounds, 100% (1 published); 20%, data for internal discussion only; 100% in agreement, publication being prepared; We have only conducted one GLP study and demonstrated very good correlation (has been published). Bridging data are not routinely generated since microsampling is used only during discovery/non GLP studies. All GLP studies are conducted using traditional sampling; I believe 90-100%? Not published; The concordance of PK/TK profiles was 50%(3/6). These data had been presented in the 47th Annual Meeting of the Japanese Society of Toxicology (2020); ~5 concordance studies: plasma vs. liquid microsampling; whole blood lysate vs DBS with at least 90% of PK parameters in concordance (1 publication); To date all results from microsampling studies have been published - the technique is used only in experiments with candidate drugs.
